# Sensing and Storing the Blood Pressure Measure by Patients through A Platform and Mobile Devices †

**DOI:** 10.3390/s18061805

**Published:** 2018-06-03

**Authors:** Vladimir Villarreal, Mel Nielsen, Manuel Samudio

**Affiliations:** Department of Computer Systems Engineering, Technological University of Panama, El Dorado 0819-07289 Panama City, Republic of Panama; mel.nielsen@utp.ac.pa (M.N.); manuel.samudio@utp.ac.pa (M.S.)

**Keywords:** blood pressure, mobile applications, web applications, mobile health

## Abstract

In this article, we present a platform that allows for the integration of different applications for the follow-up of patients with chronic diseases. We developed two elements: a mobile and a web application. The mobile application allows the capture and processing of vital signs for patients with high blood pressure (hypertension). This application allows for the patient to store the data obtained, provides historical information and trends of the stored measures, and provides alerts and recommendations according to ranges of measures that were obtained. The web application allows the doctor and patients to obtain updated information of the disease behavior through the measures obtained. We used different biometric devices including an efimomanometer, glucometer, scale, and a thermometer with a wi-fi connection. Through this web application, we also generated information about average measures at a given time, by age, by region, and by a specific date. The developed system was evaluated in a medical center with different types of patients.

## 1. Introduction

Hypertension is a public health problem that affects millions of people worldwide. Hypertension is an asymptomatic disease that is easy to detect, but if not treated early, can lead to serious or fatal complications. In 2010, a global disease burden study revealed that nine million people died because of hypertension, making this cardiovascular problem the world’s leading health risk factor [1]. Worldwide, approximately 40% of adults aged 25 and above had been diagnosed with hypertension; the number of people with the condition rose from 600 million in 1980 to one billion [2]. The prevalence of hypertension is the highest in the African Region, at 46% of adults aged 25 and above, whereas the lowest prevalence at 35% is in the Americas. Overall, high-income countries have a lower prevalence of hypertension, at 35%, compared with other groups at 40% [3]. The incidence of hypertension has doubled in the last five years in all social strata. Between 20 and 40% of the adult population in the Region of the Americas are estimated to suffer from hypertension. At the global level, of the people with hypertension, of the 57% that have been estimated to be aware of their condition, 40.6% receive antihypertensive drug treatment, but only 13.2% attain controlled blood pressure figures. This gap between the number of hypertensive patients, the access to treatment, and the achievement of control is accentuated in middle- and low-income countries, where 80% of the burden attributed to cardiovascular diseases occurs [4].

The President of the Panamanian Heart Foundation, Bey Mario Lombana, said that out of every three adults, one is hypertensive, “33% of the adult population suffers high blood pressure. Of these people, there is a third party who does not know that they are suffering, have not been detected. Panama, has its numbers, which are not the best” [5]. According to information from the Ministry of Health that is contained in its official website, this proportion increases with age: 1 in 10 people between 20 and 40 years old, and 5 in 10 for those that are 50 to 60 years old.

These figures are similar to those of the PREFREC (Prevalence of Risk Factors Associated with Cardiovascular Disease) study conducted in 2010 by the Gorgas Memorial Institute for Health Studies and the Ministry of Health, which indicated that 28.4% of the study participants reported a history of hypertension due to physician diagnosis, and 24.1% had blood pressure values that were classified as hypertension [6].

As a consequence of this disease, patients become dependent on medicines and medical care. The research and development of this study aimed to improve the quality of life of each patient with hypertension that depends on constant measurements and frequent check-ups. In addition to making use of mobile technologies to reach the largest proportion of the population possible in order to help in the early detection of this disease, a web and mobile platform are being implemented to manage patient data in the country’s health center.

Users need tools that facilitate the development of their activities through the adequate data management of information, including blood pressure, weight, height, and body mass index. Correct management allows people to change eating habits and lifestyle through the visualization of the historical measurements that were obtained over time. These historical data allow for people to view trends according to behavior during the last days, weeks, and months. 

In the health system, doctors can access each of the patients registered in the platform, so they can visualize the history of the measurements that were obtained. This enables the generation of a prevention plan more appropriate to each patient’s characteristics and tendencies. To maintain the security and integrity of the data, defined by local laws, the patient must give their consent the first time the doctor wants to access their medical record and history. Once the doctor has access, they can add recommendations, evaluate patient alerts, and add new measures.

This solution focuses on the prevention of hypertension problems, based on the self-management of data by the patient, correct follow-up by doctors, and the evaluation of the behavior of the population by the health institutions in the country. The assessment that is generated in this research confirms the need for medical data management tools that directly impact human activities. The greater use of the platform will allow for us to have more precise indicators about the population and the associated risk factors.

In this paper, we present a platform that allows for patient self-control of chronic diseases using biometric devices. First, we introduce the problems. In Section 2, we present some related works for different technologies. Section 3 explains the general functionality of the project, and in Section 4, the platform architecture is explained through the model and different processes are developed. Section 5 presents an evaluation by users in a medical center, and finally, Section 6 outlines our conclusions and possible future work. 

## 2. Related Work

Relatives today are in charge of the care of their dependents. However, given societal changes that have resulted in increased working hours, fewer days at home, and a lack of family organization make this model unsustainable in the medium term. When patients are in a care setting, they are often isolated from their social environment: a situation that greatly complicates monitoring or the patient’s privacy. The monitoring of people who are at risk is completed using equipment that is not mobile, or transportation becomes complicated.

The lack of staff in residential complexes means that not all patients can be actively attended, since usually, to reduce costs, the ratio between the number of patients and medical staff is very high, and this decreases individualized attention to patients. The medical control solutions that were achieved through technology have facilitated the remote and mobile monitoring of patients, improving the patient quality of life [7].

We defined four elements required in all mobile-Health (m-Health) applications independent of the disease: generic, adaptable, remote, and mobile. Applications must be generic so that they can be used for multiple diseases. Each disease must be cleanly implemented without modifying the modules already developed. Applications must be adaptable so that services can be tailored to each type of disease and personalizes the user’s characteristics, allowing for the interaction to be more transparent. Applications must also be remote so medical staff can monitor the data obtained in a non-intrusive manner by mobile patient biometric devices. Applications must also be mobile because development is based on the integration of small, portable, and wireless devices. 

Some studies addressed the problem of patient medical follow-ups. Bosch [8] proposed a system that provides sanitary control for patients to reduce the possibility of hospitalization. The problem with this proposal was that the patient had to interact frequently with the mobile device. 

Bengtsson et al. [9] described the relevant aspects of hypertension and hypertension treatment for use in the development of an interactive mobile phone self-report system. AUTHOR et al. [10] developed a mobile application that had the ability to communicate with blood pressure dispositive via Bluetooth. Various telemedicine solutions are based on developments for mobile devices [11,12,13,14,15]. These solutions are focused on topics, such as measured blood-pressure, heart rate, and healthcare in general.

In addition, Ryder et al. [16] presented an important tool to evaluate the health of patients suffering from chronic diseases affecting their mobility such as multiple sclerosis (MS), Parkinson’s disease, and muscular dystrophy through evaluation when walking. Our proposal maintains the background behavior that is suggested by Ryder, meaning that patient monitoring application is executed without interfering with the daily use of the mobile device, ensuring that the functionality is transparent to the user and minimizes interaction. Other proposals [17,18] integrated the Internet in the process of patient follow-up, which limits the patient’s free mobility.

In the literature, the developments in hypertension data management have focused on the development of applications for mobile devices. In our study, we focused on the development of a Web platform that offers services to patients, medical specialists, and health institutions. This platform receives data from the mobile application, which is the main platform for patient interaction. With these elements, we offer a data management space for all those involved in the process of monitoring patients with blood pressure problems. However, the evaluated solutions and developments do not offer mechanisms for the generation of indicators (patients by gender, age, demographic region, blood pressure range, weight, body mass index, etc.) that allow for health institutions to know the state of the population, and thus be able to generate prevention plans for specific sectors, based on the indicators. Our proposal seeks to minimize this interaction with technology, avoiding intrusion into the daily life of the patient.

## 3. General Functioning

The AmIHealth platform uses both Web and mobile technologies that are commonly used in Panama, based on the characteristics required for the automatic control of arterial hypertension, adjusted to the international guidelines for the classification of blood pressure [19]. AmIHealth was developed in the PHP language for the Web, environments using the Laravel framework, and Java for Android mobile devices [20]. Information storage is performed by integrating the database manager MariaDB with an encryption process of the information that is gathered by the platform.

The system provides a means of communication between patients and doctors, to shorten distances and reduce consultation times by supporting and facilitating decision-making. This system has a mechanism for describing blood pressure measurements, following the algorithm offered in “The Seventh Report of the Joint National Committee on Prevention, Detection, Evaluation, and Treatment of High Blood Pressure” (JNC) and the “European School of Hypertension—European Society of Cardiology” (ESH/ESC), to determine the category for each blood pressure measurement that is entered into the system.

In Panamá, according to the National Guide of Arterial Hypertension of 2004, arterial hypertension is considered as a blood pressure greater than or equal to 140/90 mmHg, and for diabetics and nephropaths, a blood pressure that is greater than or equal to 130/80 mmHg. All of the measurements below this range are considered as normal blood pressure.

After considering the proposed classifications from more recent studies, such as the seventh report of the Joint National Committee on the prevention, detection, evaluation, and treatment of high blood pressure, and the European guidelines for the management of hypertension, we decided to use the classification of the Latin American Consensus on Arterial Hypertension for our platform, where hypertension is diagnosed when blood pressure is greater than or equal to 140/90 mmHg, as shown in Table 1.

As such, the platform uses a visual mechanism to define ranges in green, yellow, and red color to indicate normal, precautionary, and risk measures, respectively, using the traffic light analogy.

## 4. Platform Architecture

Due to the need to reduce costs in the production and complexity of software, we chose PHP as the programming language. This language was chosen because it learns and integrates new libraries easily, and it minimizes the added cost to the project. PHP is a free, open language, with the largest active programming language community. According to Top Programming Languages of The IEEE Spectrum, PHP is in eighth position of the top 10 Web programming languages [21].

Development in a framework must be supported to facilitate the integration of our database and ensure the possible aggregation of new modules for patient care. Based on this, we implemented the Web platform in a framework called Laravel [20]. Laravel is flexible and adaptable to much architecture, not only to Model-View-Controller (MVC) architectures. Laravel allows modular aggregation, and has an extensive system of packages and drivers with which the functionality can be extended in an easy, robust, and secure way.

Laravel facilitates data management using Eloquent [22], which is an ORM (Object-Relational Mapping) based on the active record pattern, facilitating interaction with databases and compatibility with the vast majority of databases on the market today. Laravel allows for the easy and safe migration of data. Other advantages of Laravel are that it allows for the creation of robust and complex queries, facilitates handling routing of our application, and generates URL-friendly control and auto-updatable links, which simplifies website maintenance [23].

**Figure 1:**
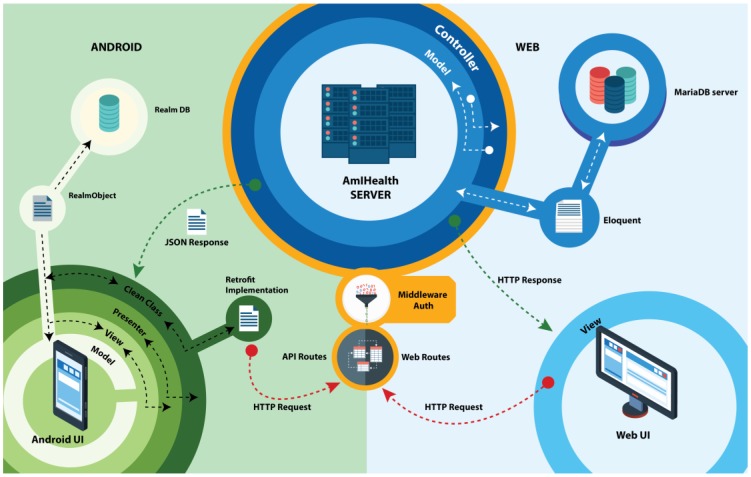
Overview of the platform architecture.

The platform was built under the Client-Server design architecture with a vision of two types of users. These clients are classified as Web clients and mobile clients, as shown in Figure 1.

The system server exposes access services to the platform through two types of routes: routes for Web clients and API Rest access routes, for access through the mobile application. All of the requests for access to data and information through the routes must be authorized and authenticated by the middleware.

The route system of the Laravel framework allows for the implementation of filtering and grouping of access routes to the system, redirecting HTTP requests to functions of the different controllers created in the platform. As such, access to information can be organized by filtering access roles, and in turn, composing the views of the platform in the Web environment. Unlike access to the web platform, the AmIHealth system exposes access to information for mobile applications through the composition of routes that are dedicated to addressing these requests.

The access route for information access for mobile applications, or API Routes, also serves HTTP requests but when the requests are answered, it is returned in JSON format. The JSON response is written and modelled to standardize the response that contains the information requested and facilitate information access by any mobile application duly registered and authorized to obtain access.

### 4.1. Authentication Process

The management of biometric or clinical electronic information in Panama is regulated by Executive Decree No. 1458 of 6 November 2012; by which, Law 68 of 20 November 2003 is regulated. This decree regulates the rights and obligations of patients in terms of information of free and informed decision [24]. Article 53 establishes that health centers, public and private, are obliged to organize, maintain, and administer, by conventional or electronic means, the clinical files of patients and ensure the integrity and the confidentiality of the information contained within. Based on this, the patient controls their information. Only if the patient grants the pertinent permissions can the medical body that was involved in the platform access the patient’s information.

The protection of data is essential for this type of application, since the information handled is medical and personal in nature. This information is related to patient data, which is why the platform does not allow for external connections without an application key or token. This token consists of a string of characters generated by the access classes and is associated with a single user.

Middleware is a convenient mechanism for filtering HTTP requests that enter the application. For example, Laravel includes middleware that verifies that the user of the application is authenticated. If the user is not authenticated, the middleware will redirect the user to the login screen; however, if the user is authenticated, the request is approved in the application [20]. This authentication process is performed for each user. Once registered in the platform, this user becomes a patient.

**Figure 2:**
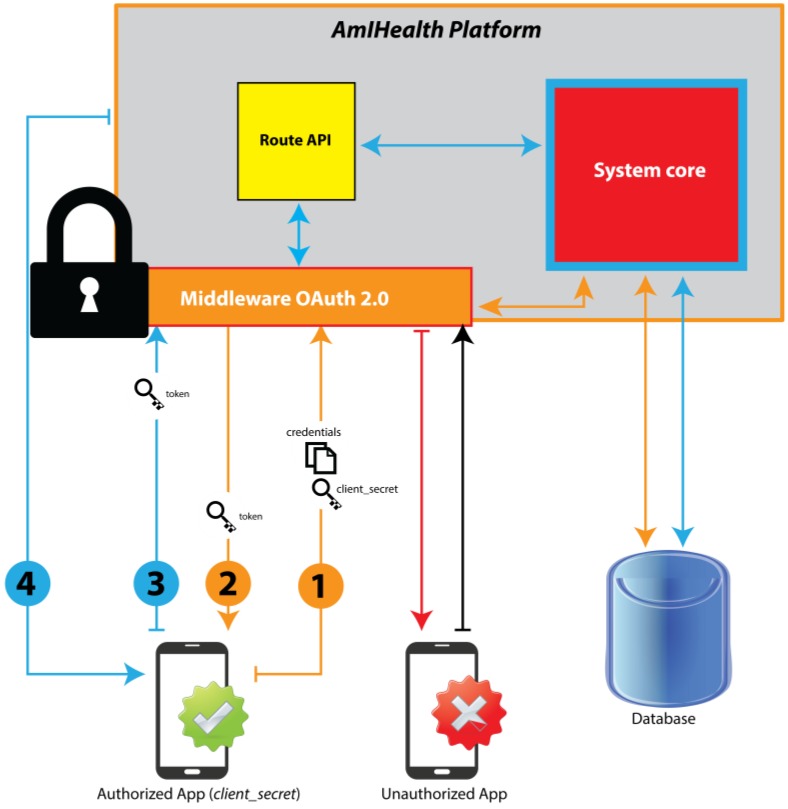
Operation of the OAuth 2.0 middleware.

The doctors can access the data of the patients registered in the platform with prior patient authorization. To access a patient’s data, the consent of the patient is necessary, so the platform verifies if the user exists when logging in from a mobile device. If so, the platform sends a query to the application, asking whether the user has allowed for access to their information. Only the patient can grant permission to access their data. However, every doctor who uses the platform to monitor and help control a patient’s hypertension can register the doctor-patient relationship in the system, so the system automatically grants said permissions to the doctor. The patient registered by the doctor must provide adequate information to comply with the registration process, such as via the email where the platform sends the patient the activation link of their registration for the platform. Figure 2 shows the authentication process of the platform through the OAuth 2.0 middleware.

When requesting services from the platform, the user must first provide their authentication and authorization credentials that will be evaluated by the access middleware. Then, the middleware consults the information that is provided by the user and compares it with the database, and then responds to the incoming request.

The use of the platform for the API Rest service clients is restricted by Laravel Passport, which provides a full implementation of the OAuth 2.0 server for the platform.

The application authorization process through Laravel Passport is as follows:

(1) Requests to the server must be made through the applications registered in the platform, which have access keys to make requests through the HTTP protocol.
(2) The key is evaluated by the middleware to confirm that the request is from an access point, Web, or authorized mobile application.
(3) If the origin of the request is not signed by a registered key, the middleware does not allow access to the request and it sends an error message of origin.
(4) If the request is signed, the middleware proceeds to distribute the request through the route class, Web or API Rest, depending on the request.

**Figure 3:**
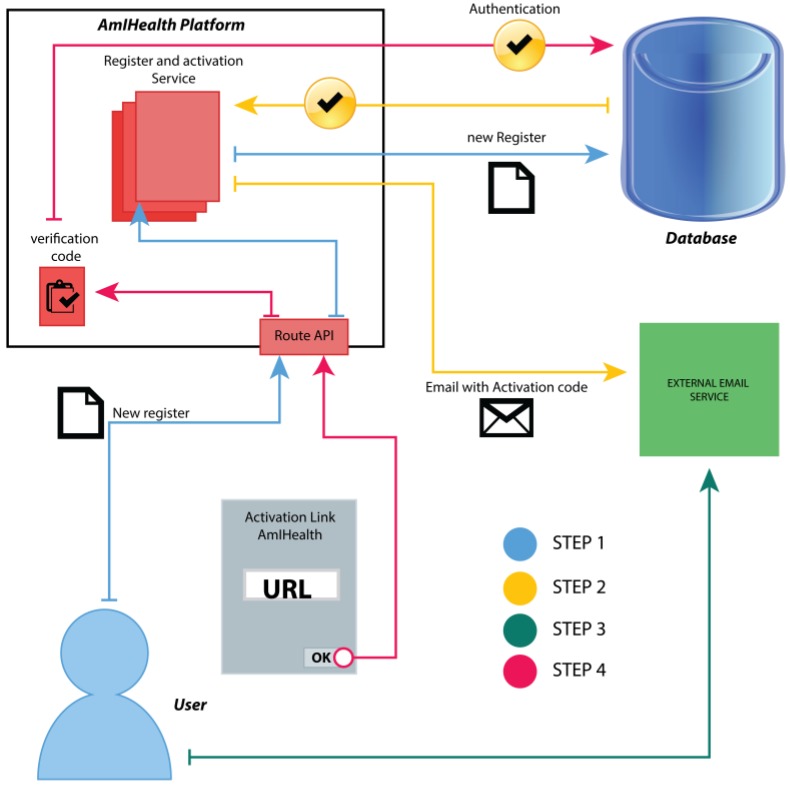
Authentication and authorization process.

The user must log in with their email and password. If there is a previous registration, then the platform provides the mobile application a validated token through the OAuth 2.0 middleware, as shown in Figure 3, and the application keeps this token for access the information located on the platform.

When the user successfully accesses the platform, they must give their consent for the use of their information in the mobile application. When delivering said permissions, the application maintains the token that will be necessary in each request that is made of the application to the platform.

### 4.2. Architecture of Clients

The platform can be consulted by different clients developed in different programming languages. So far, the platform has been integrated into the service: the Web Client and the Android Client.

#### 4.2.1. Web Client Architecture

The Web platform was developed with the Laravel framework, which uses the Model-View-Controller (MVC) pattern. The MVC is an architecture pattern that separates an application into three main logical components: the model, the view, and the controller. Each of these components is built to handle specific aspects of the development of an application. The MVC is one of the most commonly used industry standard Web development frameworks to create scalable and extensible projects [25].

**Figure 4:**
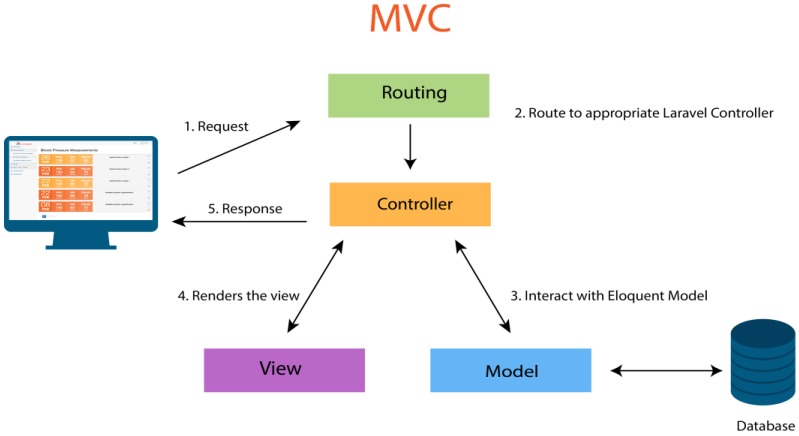
Model-View-Controller (MVC) software architecture pattern.

In Figure 4, you can see the operation of this architectural pattern in the Laravel framework. This process is divided into the following steps:

(1) The client sends a request from their browser.
(2) The route invokes the appropriate driver to handle the client’s request.
(3) The controller interacts with the Eloquent Model to obtain the information from the database.
(4) The view receives the information, renders the view, and then sends it back to the controller.
(5) The controller sends the view as an answer.

The Laravel framework includes a relational data mapping system called Eloquent ORM [26], which facilitates the creation of models. This ORM is based on the Active Record Pattern [27] and its operation is very simple. The use of Eloquent is optional, since it also has other resources that allow for interaction with the data, or specifically the creation of models. Eloquent includes a template processing system, called Blade [28]. This template system allows for the creation of much cleaner code and reduced syntax in the views.

The use of Eloquent facilitated the creation of classes of data models. With the inheritance obtained from the Eloquent class and all its ORM features, data relationships can be accessed more quickly without the need to create the SQL statements of each relationship. On the other hand, the general management of the objects that are contained in the array of the models allows for a coherent flow of the data from the database to the controller; and later, to the views of the application, allowing the grouping of these data and the immediate generation of queries on the same array of these models. Eloquent also allowed for controlling the entries of the data and its output, thanks to the data matadors that were used to transform the data to our data models.

#### 4.2.2. Dashboard

**Figure 5:**
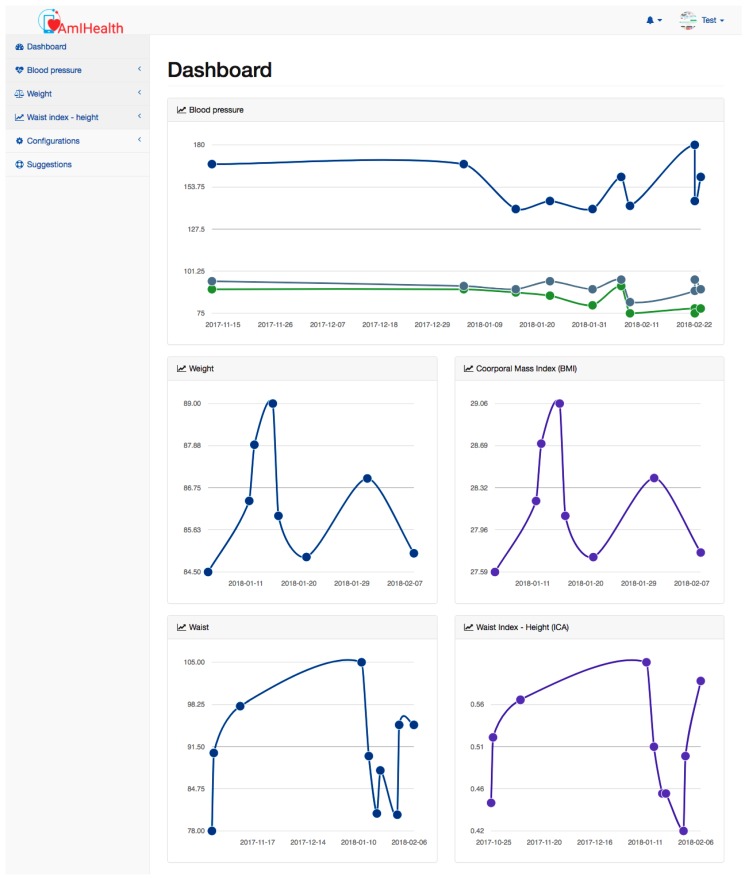
Dashboard.

The patient’s dashboard shows the most recent measurements of blood pressure, weight, and waist. These measures are presented graphically to create an easy and simply visualisation for the patient. In addition, the dashboard has a sidebar that shows the different actions that the patient can perform, such as adding a new measurement, and listing and graphically presenting blood pressure measurements, as shown in Figure 5.

The dashboard functions as a statistical and visual tool for the advancement of the patient, but for its understanding, the patient must be aware of how the blood pressure measurements are qualified and characterized. The dashboard is designed so that the user can quickly observe the historical measurements. The patient standing on a point of the graph can observe the history of systolic and diabolic blood pressure, pulse, weight, body mass index (BMI), and other measurements. In addition, the doctor can graphically observe the progress of the patient, which allows them to offer a tool to visually educate the patient. The interpretation and follow-up of the measurements and graphs begins with the consultation of the doctor, who explains how to evaluate the results in the historical graphs.

#### Blood Pressure Module

**Figure 6:**
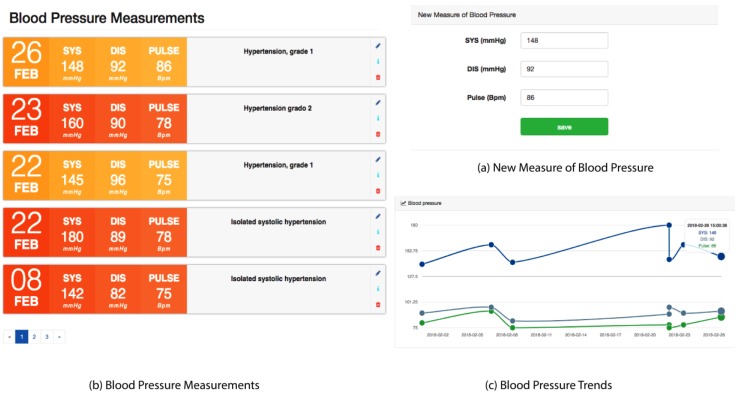
Blood pressure module.

In Figure 6a, New Measure of Blood Pressure, the view captures a new blood pressure measurement. The patient must manually enter the systolic, diastolic, and pulse values in the corresponding fields. For systolic and diastolic pressures, the values should be given in millimeters of mercury (mmHg), and for the pulse, in beats per minute (bpm). It is recommended that the patient use certified tensiometers for more accurate measurements.

The patient can list their blood pressure measurements, which are shown chronologically as in Figure 6b, Blood Pressure Measurements. This list of arterial pressures shows the date, systolic pressure, diastolic pressure, pulse, and the classification of the measurement, according to the ranges that are defined in Table 1. In addition, this view has buttons to edit a measurement, see information on the classification, and delete a blood pressure measurement. Also, as can be seen in Figure 6c, the patient can graphically and chronologically view the latest blood pressure measurements.

The automated capture of blood pressure measurements are facilitated by the implementation and use of biometric technologies combined with the accessibility of technologies, such as Bluetooth and other communication technologies. Many of these technologies are proprietary or closed protocol, which limits the capture of data from these devices. Conversely, the public health system in Panama limits the use of blood pressure collection devices to a certain type of device, with manual devices predominating. Based on the reality of the Panamanian population, who do not have a biometric device with these technologies, the insertion of data in this first version, is performed manually, and thus does not exclude people who do not have the latest generation devices. For future works, automatic readings will be included according to device availability that facilitates this process.

#### Weight Module

Obesity or overweight is one of the risk factors that increase the probability of a patient suffering from pre-hypertension or high blood pressure. Overweight and obesity refer to a body weight that is greater than what is considered to be healthy for a given height.

This weight module is very important for the platform, since it allows for the recording of historical weight measurements of the patient, using the classification of the World Health Organization (WHO) and the nutritional status according to the Body Mass Index (BMI) [29].

**Figure 7:**
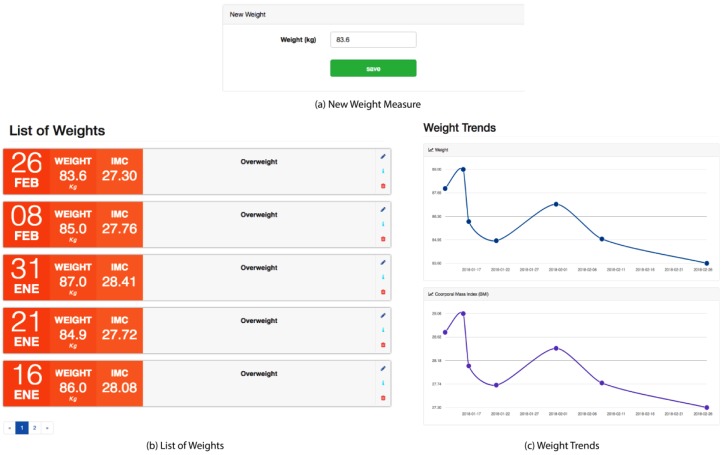
Weight module.

As can be seen in Figure 7, the patient can insert a new weight measurement, and then the system calculates the BMI using a mathematical ratio that associates the mass and the height of the patient. The mass is expressed in kilograms and the square of the stature in meters squared. The value obtained is not constant, but it varies with age and sex. In the case of adults, BMI has been used as one of the resources to evaluate nutritional status, in accordance with the values that are proposed by the World Health Organization (Table 2).

The patient can list weights, which contain the date, weight, BMI, and classification of the measurement. In addition, the trend in weight and BMI can be viewed in graphic form for a better understanding of progress.

#### Waist-to-Height Ratio Module

This module is an alternative parameter of the body composition measurement, as BMI was used as a body composition methodology. Other more widespread alternatives are available that, according to stages, provide guidelines for evaluating cardiovascular risk and nutritional status, such as the Waist-to-Height Ratio (WHtR) or Waist-to-Stature (WSR) [30]. These measurements are the quotient between the circumference of the waist and the height, both being measured in the same units. The higher the WHtR value, the greater the risk of obesity and cardiovascular diseases. A WHtR value above 0.5 denotes a considerable risk. According to a recent study [31], WHtR has become the most prominent and reliable tool other than BMI for detecting the risk of heart attack, myocardium, or death.

**Figure 8:**
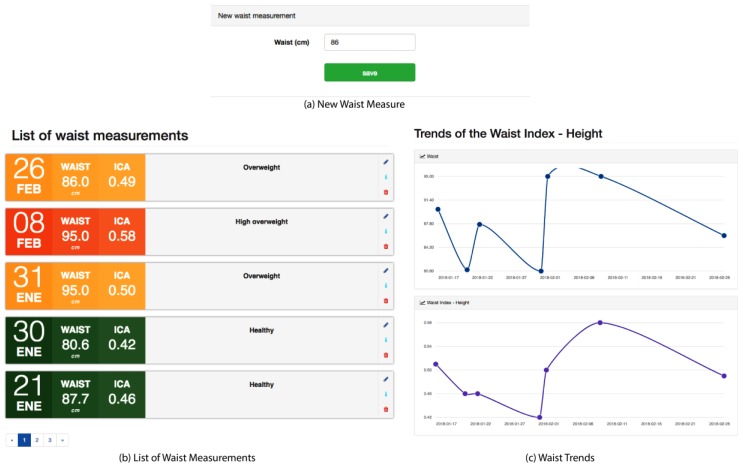
Waist-to-Height Ratio module.

Figure 8 shows the interfaces of this module where the patient can insert a new waist measurement that must be entered in centimeters. The system registers the new measurement, calculates the WHtR using the classification shown in Table 3, and shows these measurements in a chronological list. This list contains the date, waist measurement, WHtR, and the classification of each measurement. In addition, the patient can graphically see the trends of the waist and the WHtR.

#### 4.2.3. Android Client

**Figure 9:**
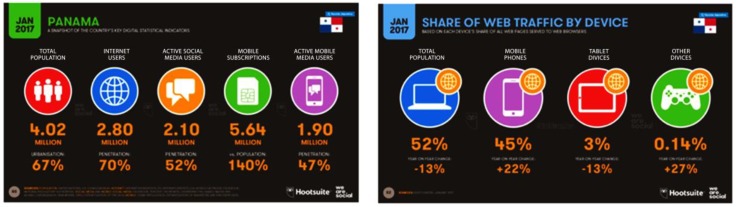
Statistics in Panama on telephony and Internet connection [17].

Mobile technology has evolved so rapidly and has proliferated among people in each country, such that there are more mobile phones than there are people. In Panama alone, of the 4.02 million inhabitants, there are 5.64 million mobile subscriptions, representing 140% as compared to the population. Notably, 2.80 million Panamanians, or 70%, are Internet users, as can be seen in Figure 9.

With respect to the Internet, 52% of users connect through personal computers, 13% fewer when compared to previous years, and 45% access the Internet using a mobile phone, highlighting an increase in 22% over the prior year [33].

Many applications exist around the world that millions of users download every day. Within the applications for the monitoring and control of hypertension, companies that are dedicated to the health sector and self-care of patients are well known, such as Nokia [34], who has a division that makes biometric devices to capture data using mobile technology.

Despite the fact that smartphones are popular in Panama, few applications developed in our country are available for purchase, and there are no published applications that allow Panamanians to track and control their blood pressure.

#### Mobile Client Architecture

The architecture of the mobile application enables the analysis of the effectiveness of the design against the requirements, which allows for considering new modular alternatives to make changes in the software, minimize the transition, and reduce the risks that are associated with its construction.

The architecture of the system is based on components following the evolutionary process model that was designed with the Model-View-Presenter (MVP) and clean pattern. 

The MVP pattern is a derivation of the design pattern of MVC interfaces that are commonly used for Web development [35]. The pattern is defined in three layers: model, which refers to the classes that define the data and the structure; view, which is the user interface with a passive domain of the application’s business logic; and, presenter is the class responsible for managing the flow of data, requests for views, and transactions with data models. Presenter is the intermediate layer responsible for the logic of the business.

Clean Architecture [36] was developed due to the need to implement simple architectures, given the overwhelming amount of errors in software architecture and the problematic patterns implemented in the projects of the creator. This architecture defines clear objectives:

(1) Independence of frameworks: Architecture should not be linked to or depend on a framework. When define this item, the developer can dominate the framework and not vice versa.
(2) Testable: Business layers should facilitate testing independent of the user interface, the database, the server, or any external agent to the layers that are present in the software.
(3) Independence of the User Interface (UI): Independence allows the UI to be changed without consequences in the operation of the software.
(4) Independence of the database: The databases can be changed without having to considerably vary the code.
(5) Independence of external agents: The business rules or logic of the software business should always be isolated from the “outside world” and not be affected by changes in the surrounding classes.

**Figure 10:**
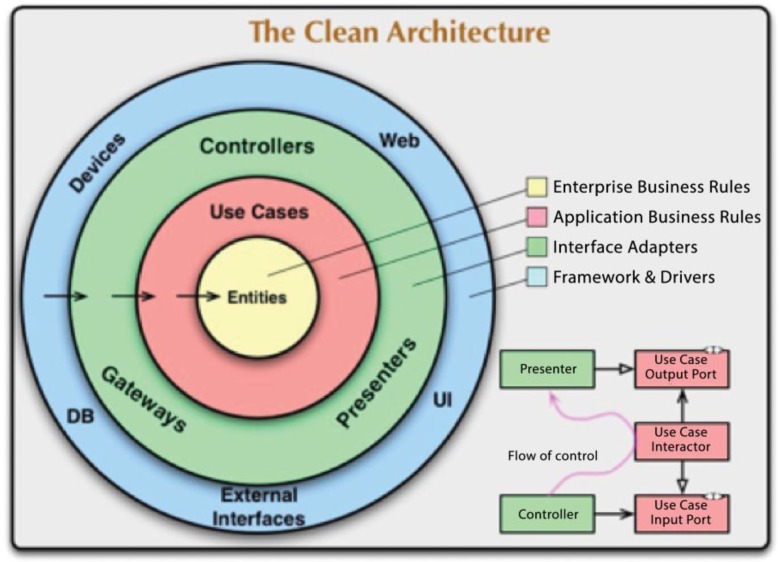
Descriptive diagram of clean architecture [36].

In Figure 10, the arrows indicate the dependence of the architecture. The layers of the architecture are represented by different colored circles to identify each layer in the architecture. A greater distance for the center of the circle denotes a layer of higher level abstraction in the software that was implemented as mechanisms and internal policies.

The dependency rules indicate that the software only knows and has access to the next internal layer. That is to say, that an entity will not simultaneously know its Use Cases and who makes use of its properties. Entities are responsible for representing the business rules in the software that implements the architecture. They are represented by the objects that encapsulate the highest-level layers and are the least exposed to change. Cases of use are those that coordinate the flow of information, accessing entities, and providing information to the upper layers. The modification of this layer must not affect the internal layer (the layer of entities), nor should it be affected by external changes. This layer contains the adapters that transform the information that is granted by the use cases to the necessary format to elevate it to the external layers of the architecture, including the database or the user interface. The purpose of this layer is to disintegrate the dependence of the internal data models of the external one. External agents compose the outermost layer of the architectural representation of the architecture. It is the layer that is the most exposed to changes, since it contains all external agents, such as databases, user interfaces, and server. The classes must be defined for specific issues, meaning they will only receive and display data if it is the user interface. The layer will save the data, if it is the database; or it will send the data, if it is the server class.

**Figure 11:**
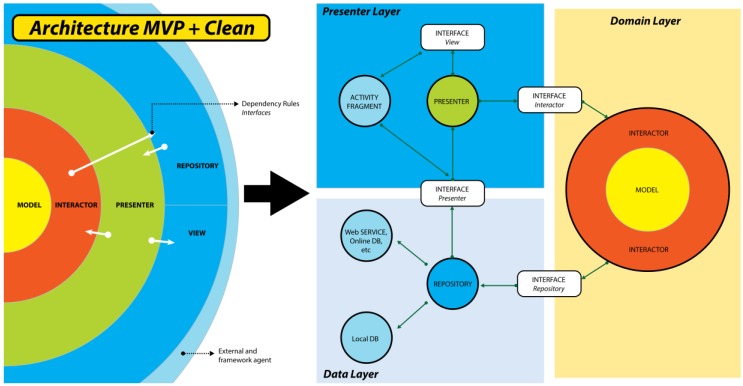
Model-View-Presenter (MVP) architecture + Clean.

To implement this architecture for the development of Android applications, describing and considering the needs and problems that Android experiences as a mobile operating system is necessary. There are well-defined hardware restrictions and methods to implement software. As a consequence, applications become dependent on implementations of databases and Web services that may vary [37]. Figure 11 shows the implementation of the MVP with clean architecture in the Android application. 

To adapt the clean architecture to the needs of the project and to organize the development project, the classes were separated and were grouped into three layers: the presentation, domain, and data layers.

The presentation layer was limited to the interaction of users, modeling each action in the main thread of the application, and it is responsible for receiving and displaying information to the user. The presentation layer includes the classes “View”, “Presenter”, XML (eXtensible Markup Language) of the UI, and the interfaces to allow for communication between the presentation classes and the views. Next, the classes that conjugate the presentation layer are detailed. The view layer is comprised of classes that are dedicated to user interaction with the application. This layer is contained by the layouts.xml and the activity.java. Each action allowed to the user in the user interface is programmed in the activity as an event and will be attended by the presentation layer. The presenter is the class in charge of managing the cases of uses duly described in the user interface, meaning every action that the user executes in the view must generate an action that must be handled. The presentation layer is responsible for managing the actions directing the process flow. The presentation class retains the logic of transforming the information into view, as well as the control of actions toward the domain and data layers.

The Domain layer is the gearing of the information in the application; it is the layer that is responsible for business logic as it describes each use case deriving an action from the repository class. This layer integrates the repository interfaces to execute the actions requested by the provider, distributed by the domain. The domain layer contains the interactor and the model. The interactor is the distribution class of the use cases; this class is responsible for transforming the user’s requests through the presenter. They are an extension of the entities in the business logic, defining the action to be executed in methods for each use case. In the model layer, any entity that acts in the application using the most descriptive and simple process is represented. The object classes or “Plain Old Java Object” (POJO) [38] were used to contain information and to facilitate the flow of the same through the classes that require them.

The data layer concentrates all kinds of data and the methods of inserting, editing, obtaining, and deleting data, known as CRUD (Create, Read, Update, and Delete). The data layer is in charge of all data access. The data layer is comprised by the repository classes. The repository class is responsible for managing access to data, defining all the access routes to information as well as the routines that are necessary for the storage of information.

The combination of MVP with clean architecture helped the development of the application, facilitating the integration of new code due to the distribution of the layers and the handling of the dependencies, facilitating the tests. In addition, this combination allowed for the creation of new independent modules to be easily added to the project. With the implementation of MVP with clean architecture, as development architecture, teamwork was facilitated.

To achieve dependent communication following the guidelines of the clean architecture, the relationship dependency outlined in Table 4 was created. In this table, the dependencies where the interfaces were used (Figure 11) to apply the dependencies are shown.

Figure 11 graphically depicts the class distribution and the applied operation based on the MVP with clean architecture. Next, each of the characteristics of the application and its definitions are described in detail to understand its operation. These characteristics correspond to the main modules of the application.

**Figure 12:**
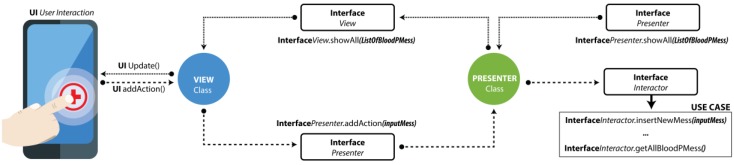
Presentation layer data flow.

Figure 12 depicts the flow of information through the presentation layer in the implemented architecture when the user interacts with the view. In this case, the layer inserts a new blood pressure measurement, and the view contains an instance of the presentation class, which in turn, implements the presenter interface by which the domain layer is requested to execute the action of insertion.

In the domain layer, the use cases of the application module are represented as methods that are part of the interactor class. In the presenter, an object of the interactor class is instantiated, which implements the iteration interface. Through this interface, the insertion action can be executed.

**Figure 13:**
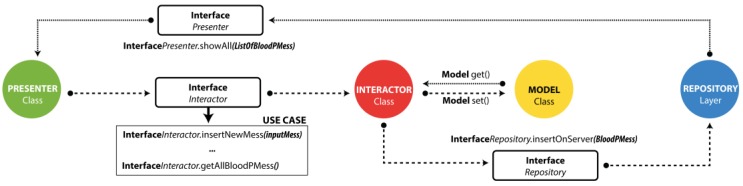
Domain layer.

Figure 13 depicts the convergence of the presenter layers. In the example image, the continuation of the request that is sent by the user through the view is displayed. That request is now sent to the domain layer where the iteration class distributes the process flow based on the use cases, and then the event is communicated to the data layer. Throughout the flow of data and requests between classes, the use of the data models or the POJO of the application is always available, which is the blood pressure model (HTA measure) in this case.

#### Interface Design

To allow patients more self-control of their condition, the application had to be based on the principles and guidelines of the design of the user interfaces that are suggested by Pressman. The application is based on Google’s new design language, Material Design. This new language is part of Android since version 5. Material Design unifies the space reacting to the movement, since it provides meaning to the user, with intrepid, graphic, and intentional elements [36].

In the analogy of the functionality of the platform, the concept of modularity was applied to the design, where each module has a specific functionality within the application. Due to this, the design of the interfaces is important. Accustoming the user to an interface facilitates the use of each function, with the use of icons and colors distinguishing the buttons of another object.

#### Blood Pressure Module through the Android Client 

Addressing arterial hypertension is the main objective of the platform. Managing, storing, and evaluating blood pressure measurements are the basic characteristics of this module. The blood pressure module aims to facilitate the management of patient or user biometric blood pressure information. It also allows for the information to be observed graphically through time and it shows the details of each measurement that are stored in the system.

#### Evaluation Process of Blood Pressure Measurement

**Figure 14:**
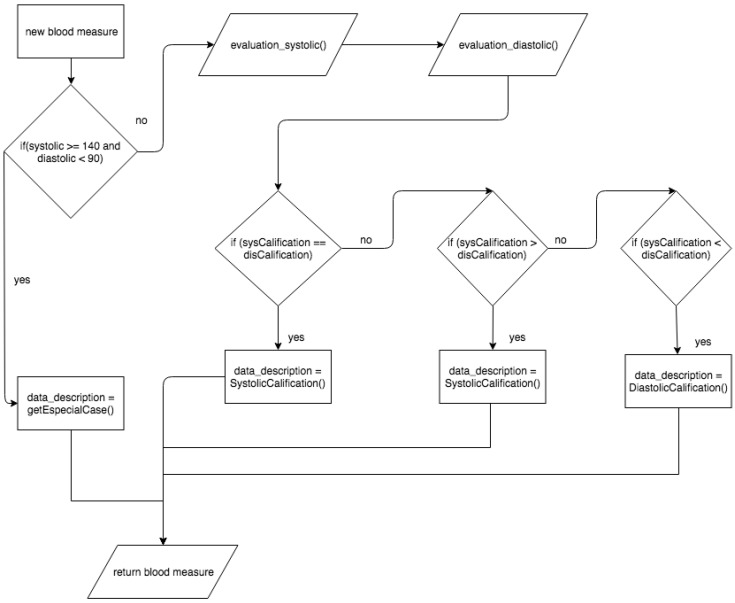
Process of qualifying blood pressure classification.

When the user enters a new pressure measurement, it must be evaluated to be qualified based on the qualification reference table being used (Table 1) within the platform. As shown in Figure 14 the evaluation process starts by consulting the blood pressure object that counts the systolic and diastolic measures, and then the values are consulted, determining special cases, such as isolated hypertension. In this case, the value is added to the object and the description is named in the hypertension qualification reference table. If there are no special cases, the systolic and diastolic measures are evaluated separately. 

The evaluation processes return a new object within the algorithm with qualifying ranges of each measurement, which are then evaluated to determine the predominant rating. Notably, each measurement is evaluated and qualified separately because, regardless of whether it is systolic or diastolic, if one of the measures qualifies as a hypertensive measure, then the measurement in general is hypertensive [39].

When entering the blood pressure measurements, they are evaluated and qualified, and the user can observe the new measurement distinguished by the color based on the category in which it was evaluated within the reference table used. The user can access the measurement details by blood pressure to the extent that they wish to analyze (Figure 14).

**Figure 15:**
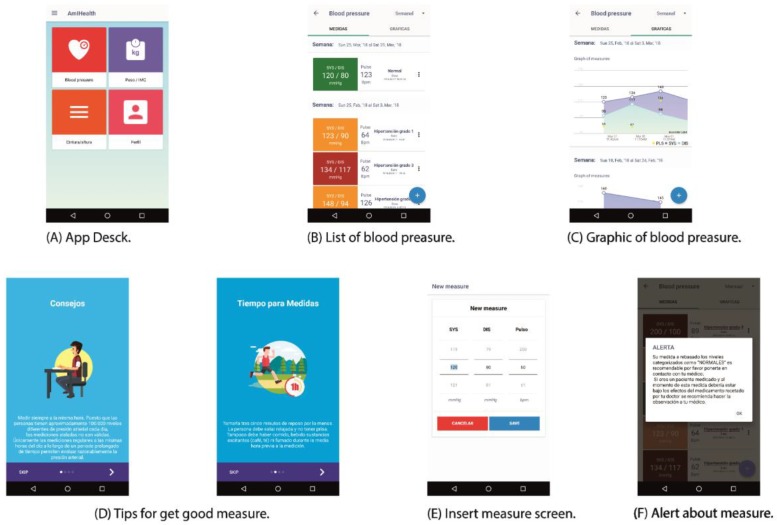
Blood pressure module through the android client.

The blood pressure process in the mobile application is not dissimilar from the web process, but unlike the web, the sidebar of the menu is hidden. However, the user is shown the menu of the care models (Figure 15A). When entering the blood pressure module, the user is shown the list of measures (Figure 15B). The list provides a chronological order of the measures; filtering options are available in the upper navigation bar. To facilitate the use of the application, it was segmented into tabs, the list of measurements, and the graphs (Figure 15C). The graphs show the measures that are scored to observe the weekly, monthly, or annual trend in blood pressure measurements.

When pressing the button (+), the interfaces are released as first instance. Advice is provided about how to obtain measurements with the smallest possible margin of error and thus obtain valid measurements that are closer to reality (Figure 15D). Then, a spinner is shown for the insertion of a new pressure measurement (Figure 15E). If the measurement is outside the ranges categorized as normal, the application sends a warning message (Figure 15F).

The mobile application is a client of the web platform. The mobile application shows and stores the data obtained, first locally, and later on the central server. If the mobile application does not have connectivity to the server, it can continue to offer the latest measurements that are stored by the patient, and can make new measurements, but only once it has activated connectivity will it be synchronized with the server. All the activities of adding, modifying, and evaluating measures are complete in both environments, with the difference being that the role of the doctor is only accessible from the web platform, focusing the design of the mobile application on functionalities for the patient. 

In summary, the patient can manage their blood pressure, weight, waist index, profile management, and visualize the graphical and tabular historical data of these measurements, from both the mobile application and the web platform. The mobile application stores the measurements that were obtained in a local database and then updates the web platform when it has Internet connectivity. This mechanism allows for the patient to maintain the functional application, even without Internet connectivity. Doctors can access the information of their patients from the web application, where they can follow up on the historical measurements obtained and add new measures in their medical office.

## 5. Evaluating the Blood Pressure Platform by Users

We evaluated the functionality, usability, and design of the developed blood pressure platform. Patients with high blood pressure, needing to provide follow-up on the disease, evaluated these domains. We implemented the platform in each of the Public Health Centers of the Ministry of Health in Panama. In each Public Health Center, records include people both with and without hypertension problems. Importantly, the platform focuses on aspects of prevention of hypertension at a young age. An average of 65 patients were registered daily on the platform for each visited Public Health Center. 

For this study, we randomly selected 63 people of different ages and sex who have attended a general medical consultation at either of the two Public Health Centers of these, 33 attended the first Public Health Center and 30 attended the second Public Health Center. These people attended to their basic care in the morning. During the medical consultation, these patients were added to the platform and they were taught how to use it. Once the consultation was finished, a questionnaire was provided to evaluate some aspects of the platform’s quality. We assessed the content, design, and usefulness of the platform in the provided questionnaire. The questionnaire had 20 questions that were classified into the three main criteria.

To evaluate the content criteria, the patients responded to questions that were related to the organization of the content, the use of the platform, ease of interpretation, ease of identification of the elements, and the degree of value of the submitted content. For the design criteria, they responded to questions about the distribution of visual elements, displays, interpretation, and identification of menu specifications. For the usefulness criteria, they responded to questions about the degree of usefulness of the generated graphics, tables, and degree of satisfaction with the information.

In terms of evaluation technique, a questionnaire was applied where participants responded to specific questions about how they used the application. We asked user to assess the aspects of content, design, and usefulness of the application. The patient used the application to monitor blood pressure for a certain time and then reviewed the medical control activities (graphics and table about the blood pressure, weight, height, and others). In both the first and second phases, the assessment was been provided to 33 people each time. We provided the assessment to 63 people (30 men and 33 women). The population was formed by people between 18 and 69 years old.

The patient spent 30 min in the application after their weight, blood pressure, waist, and height were measured. Then the people answered the questionnaire for 20 min. A Likert scale from one to five was established to evaluate each question, where one indicated strongly disagree, two indicated disagree, three indicated neutral, four indicated agree, and five strongly agree. 

**Figure 16:**
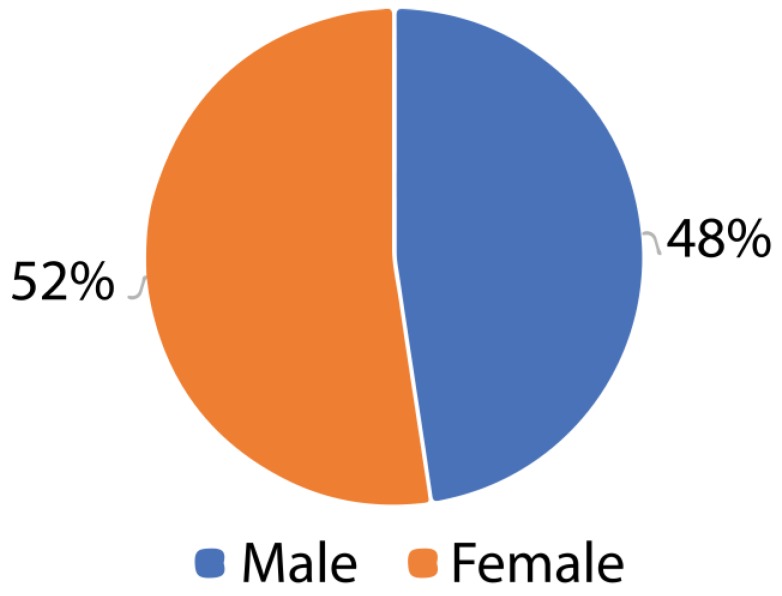
Classification of users by sex.

Figure 16 shows that most respondents were women at 52%, with 48% being men. The women showed more agility in the use of the application. They also showed more interest later in the use of the application.

**Figure 17:**
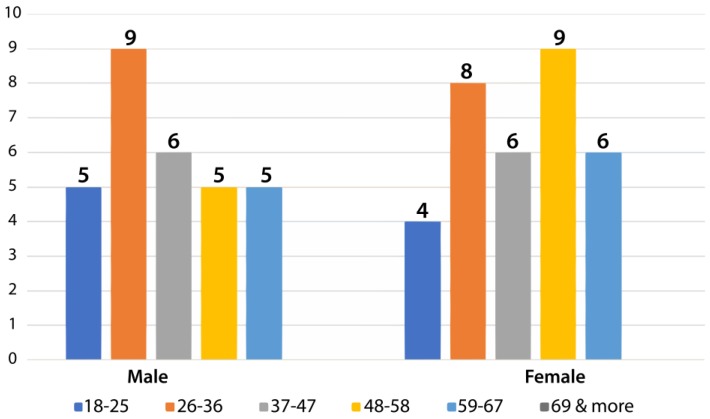
Classification of user by age.

During the study, the population was distributed equally among the different age groups requested, with nine participants aged 18–25, 17 aged 26–36, 12 aged 37–47, 14 participants aged 47–57, and 11 aged between 58 and 68 (Figure 17).

Measurements were recorded for blood pressure (systolic and diastolic), weight, height, and waist circumference for each participant. These elements are important since they are considered risk factors for suffering from hypertension: height, weight, and waist. 

Most of the participants were in normal to high light ranges, in comparison with their weight and stature. Those people with high waist and weight measurements had high blood pressure. After the measurements, the participants completed a 20-item questionnaire to evaluate the platform, classified into design, usefulness, and content of the blood pressure data management platform. 

### 5.1. Evaluating the Content Quality 

For this paper, we wanted to obtain information and opinions of the users related to the content of each interface. We analyzed this aspect based on organization of the information, graphic and text, completeness, and clarity of the information. Also, the ease of navigation, location of messages, and screens and the information quality for the user were evaluated. 

**Figure 18:**
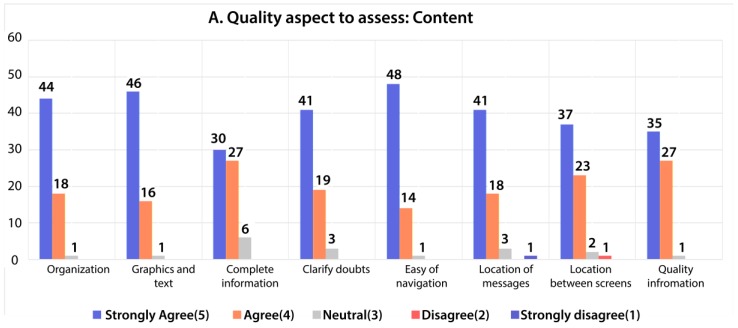
Evaluation by users about the content of the platform.

After the application was used by the users, eight questions were posed to assess the aspects of the content of the application, as shown in Figure 18. After the analysis, 63.89% of the users strongly agreed about the quality of the content of the application, text, ease of navigation, location between screens and messages, and the quality of the information. However, 32.14% valued this aspect as agreed, 3.57% as neutral, 0.20% disagreed, and 0.20% strongly disagreed. 

For each questionnaire applied, we collected information about the age of each participant. Notably, those responding with neutral, disagree, and strongly disagree were generally participants that were aged 47 to 68 years old. These people had greater difficulty using mobile devices. 

### 5.2. Evaluating the Design Quality Aspect

**Figure 19:**
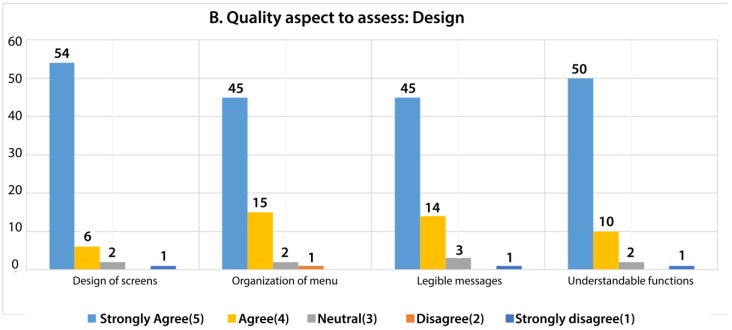
Evaluation of users about the design criteria.

In the design quality aspect, four questions were asked of each participant. We asked about the design of the screens, menu, messages, and functions, as shown in Figure 19. After the analysis, we 76.98% responded with strongly agreeing with the design quality aspect. However, 17.86% responded with agree, 3.57% with neutral, 0.40% with disagree, and 1.19% with strongly disagree. Similar to the content quality aspect, the responses that were associated with neutral, disagree, and strongly disagree were associated with those respondents that were aged 47 to 68 years old. 

### 5.3. Evaluating the Usefulness Quality Aspect

**Figure 20:**
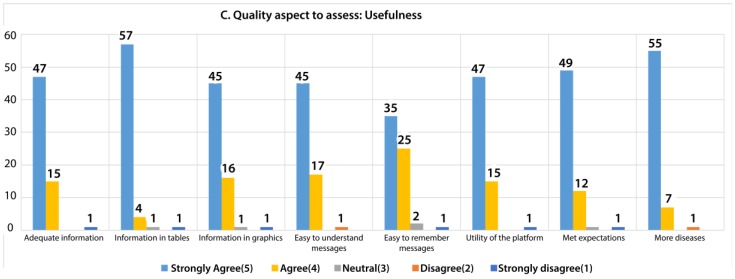
Evaluation of users about the usefulness criteria.

In the usefulness quality aspect, eight questions were asked of to each participant, as shown in Figure 20. Usefulness and the content were the highest priority in the development of the application because we wanted to provide an application that offers useful information to patients. The design was an additional element that gives the application a better look.

After the analysis, 75.40% responded with strongly agree about the usefulness of the platform. However, 22.02% responded with agree, 0.99% with neutral, 0.40% with disagree, and 1.19% with strongly disagree. Similar to content quality and content aspect, the responses that were associated with neutral, disagree, and strongly disagree corresponded to respondents aged 47 to 68 years old.

After evaluating the application in a real environment at local health center where many patients from different economic and family situations and ages, who never use of mobile devices, and especially people who never control their blood pressure through technological devices, we can assure that this first approach offers a solution that is currently being used in the Public Health Centers. 

## 6. Conclusions and Work in Progress 

In this work, we offered a solution for patients, relatives, and doctors with a web solution and a mobile phone application. We verified that patient self-control could be facilitated through the use of our developed data management platform. Patients who have interacted with the platform showed interest in managing their data daily and visualizing the progress of their disease. Based on the four elements that we considered to be necessary for a chronic disease monitoring application, our solution is generic, adaptable, remote, and mobile. Our application is generic because the platform allows for the development of new modules for new measurements without modifying the existing ones, adaptable because it adapts to the needs of each patient. It is remote because the storage, although it is local in the first step, is always updated in the web platform, and our application is mobile because a user-friendly interface for mobile was developed in terms of design, content, and use.

We based our development on a platform that allows the easy and fast integration of new patient applications. Our main goal was to promote easy daily disease monitoring for people with a chronic condition. This architecture provides continuous patient monitoring to improve the communication between patients and doctors, allowing for the generation of an automatic architecture for individual patient profiles, self-control, and education modules for their chronic disease. This was developed for the mobile monitoring of patients via biometric devices and a mobile phone. The assessment presented in this article shows that a section of the population remains that, due to ignorance of new technologies, they cannot make use of the solutions that are offered by a platform such as the one presented. However, after implementing the evaluation of the platform by the users, we realized that a large part of those treated in health centers are people between 18 and 30 years old who suffer from hypertension problems. Developing solutions like these, while considering that this age group is generally knowledgeable about existing technologies, can help to manage and organize the data generated daily, for better management and control of chronic disease. After the assessment, the women showed more agility in the use of the application. They also showed more interest in the continued use of the application.

If users have a data management system, they can better control the data generated by blood pressure, which allows for decision-making based on evidence and facilitates follow-up by health specialists. This type of solutions also allows for generating future modules based on stored information, and creating specific prevention plans aimed at each region of the country. 

Although other (proprietary) applications for hypertension data management are available, none of the presented solutions obtain data so that the health institutions of each country can generate statistics on the performance of the region. This project provides a technological solution that facilitates the prevention and management of chronic diseases, such as hypertension, based on the provisions and strategic axes that are defined in the National Plan for the Promotion of Health 2016–2025 defined by the Ministry of Health [40].

We are working on new modules to analyze risk factors that are related to blood pressure problems. This module will allow for health institutions to generate hypertension indicators according to the region of the country where the users are located. It also generates statistics classified according to the indicators and risk factors defined in the platform. We defined this project as a research initiative that allows for the management of patient data with chronic diseases.

## Figures and Tables

**Table 1:** Classification of blood pressure. Source: Latin American Guidelines for Arterial Hypertension.

ESH-ESC	Systolic (mmHg)	Diastolic (mmHg)	JNC-7
Optimal	<120	<80	Normal
Normal	120–129	80–84	Pre-hypertension
High Normal	130–139	85–89	Pre-hypertension
Hypertension			
Grade 1	140–159	90–99	Grade 1
Grade 2	160–179	100–109	Grade 2
Grade 3	≥180	≥110	Grade 3
Isolated systolic	≥140	<90	Isolated systolic

**Table 2:** The international classification of adult underweight, overweight, and obesity according to BMI. Source: Adapted from WHO, 1995, WHO, 2000, and WHO, 2004.

Classification	BMI (kg/m^2^)
Underweight	<18.5
Normal	18.5–24.9
Overweight	25.00–29.99
Obese I	30.00–34.99
Obese II	35.00–39.99
Obese III	≥40.00

**Table 3:** Waist-to-Height Ratio classification using waist circumference and waist-hip ratio [32].

Children and Adolescents (up to 15 Years Old)	Man	Women	Category
<0.34	<0.34	<0.34	Extremely Slim
0.35–0.45	0.35–0.42	0.35–0.41	Healthy Slim
0.46–0.51	0.43–0.52	0.42–0.48	Healthy
0.52–0.63	0.53–0.57	0.49–0.53	Overweight
≥0.64	0.58–0.62	0.54–0.57	Overweight elevated
	≥0.63	≥0.58	Morbid Obesity

**Table 4:** Relationship between clean architecture and dependency.

Implementation Class	Interface	Object of Communication	Response to Class
Activity	ViewInterface	Obj Prensenter	
Presenter	PresenterInterface	Obj Interactor	Interactor
		Obj ViewInterface	Activity
Interactor	InteractorInterface	Obj Repository	Repository
Repository	RepositoryInterface	Obj Presenter	Presenter
